# Granzyme B-expressing regulatory B cells share the same origin as conventional blood B cells

**DOI:** 10.1016/j.bbrep.2026.102594

**Published:** 2026-04-17

**Authors:** Steve Genebrier, Nicolas Sailliet, Samuel Bastos Serra Trinca, Hoa Le Mai, Amandine Dupuy, Marie Cornic, Richard Danger, Magali Giral, Karin Tarte, Sophie Brouard, Michel Cogné

**Affiliations:** a UMR1236, Université Rennes, INSERM, Etablissement Français du Sang, Rennes, France; b SITI Laboratory, Rennes University Hospital, Etablissement Français du, Sang, Rennes, France; c CHU Nantes, Nantes Université, INSERM, Center for Research in Transplantation and Translational Immunology (CR2TI), UMR1064, ITUN, Nantes, 44000, France; d LabEx IGO « Immunotherapy, Graft, Oncology », Nantes Université, Nantes, 44000, France

**Keywords:** B cell repertoire, Granzyme-B expressing B cells, Regulatory B cells

## Abstract

Regulatory B cells (Bregs) play a pivotal role in various dysimmune conditions. We have previously demonstrated that granzyme B-expressing (GZMB^+^) B cells are more numerous in patients with graft tolerance following kidney transplantation and that these cells display regulatory properties through GZMB. However, their ontogeny remains poorly understood.

To address this gap, we analyzed the B cell receptor repertoire of GZMB^+^ Bregs and GZMB^−^ B cells sorted from healthy donors and drug-free tolerant kidney-graft recipients.

This revealed that both subsets exhibited similar repertoire features in terms of diversity, V/J gene usage, CDR3 profile and somatic hypermutation pattern, suggesting a common origin and recent divergence. Furthermore, there was limited overlap between GZMB^+^ and GZMB^−^ clonotypes within individual donors. Taken together, although these observations should be interpreted with caution given the limited cohort size and considered primarily descriptive, these findings suggest that GZMB^+^ Bregs likely arise from the conventional mature B cell pool and represent a differentiation state shaped by environmental cues rather than constituting a distinct clonal lineage.

## Highlights

• GZMB + regulatory B cells emerge in transplant-tolerant patients via unclear pathways.
• Repertoire analysis suggests recent GZMB + B cell differentiation from GZMB- precursors, within the limits of our dataset.
• Based on clonotype analysis, GZMB^+^ B cells are more likely induced by specific stimuli than to represent a discrete lineage.

## 1 Introduction

Regulatory B cells (Bregs) are a diverse ensemble of cells that carry immunosuppressive functions. They are defined according to their ability to temper immune responses in different pathophysiological contexts [1,2]. Despite their univocal functional relevance, Bregs lack a unified phenotypic definition and their identification remains primarily context-dependent [3]. Among the various reported phenotypes, granzyme B-expressing (GZMB^+^) B cells have emerged as a distinct and functionally potent Breg subset, in both rodents and humans [[4], [5], [6], [7], [8]].

We have previously shown that GZMB^+^ B cells are enriched in the peripheral blood of kidney transplant recipients who exhibit operational tolerance (drug-free tolerant patients, TOLs), suggesting that they may play a role in regulating alloimmunity [6]. Their suppressive function is not yet fully elucidated but partially dependent on GZMB and contact between the cell and its target [6].

Whereas they may represent around 3 to 5% of total B cells in tolerant patients, they only represent around 1% of peripheral B cells in healthy volunteers [6]. For that reason, we developed a protocol to generate them *ex vivo* from total B cells using a defined stimulation cocktail comprising BCR ligation, CD40L, CpG ODN, IL-2, and IL-21, yielding up to 95% GZMB^+^ B cells after three days in culture [8,9]. Thanks to these protocols, the transcriptomic profile of these GZMB^+^ B cells was characterized in both healthy donors [10] and kidney transplant recipients [11]. These analyses revealed a distinct differentiation trajectory, shared across several Breg subsets, which diverges from classical switched memory B cells. Specifically, GZMB^+^ B cells uniquely express the transcription factor KLF13 [11]. This raises the question of whether they may represent a distinct lineage, unlike other Breg subsets such as IL-10^+^ B cells (B10) that can emerge from classical B cells at different stages of differentiation [1].

To further explore the ontogeny of these elusive cells and track a potential B cell repertoire signature, we analyzed the immunoglobulin heavy chain (IGH) repertoires of unmodified sorted GZMB^+^ and GZMB^−^ B cells from the blood of two healthy donors and two TOLs using next-generation sequencing. We then compared the key parameters characterizing B cell repertoires in the two subsets. These included V/J gene usage, diversity indices, mutation frequency, and various scores attached to the CDR3 sequence. The results revealed concordance between the two subsets, and altogether suggest that, under appropriate stimulatory conditions, GZMB^+^ B cells emerge from the conventional B cell pool.

## 2 Materials and methods

### 2.1 Samples and cell isolation

This study was performed in accordance with the Declaration of Helsinki and approved by the National French Ethics Committee: DIVAT (Données Informatisées et VAlidées en Transplantation - www.divat.fr, French Research Ministry: RC12_0452, last agreement No. 13,334, No. CNIL for the cohort: 891735 and ClinicalTrials.gov recording NCT02900040). All participants enrolled in this study signed informed consent forms. Blood was obtained from healthy adult donors (Etablissement Français du Sang EFS, Nantes, France, CPDL-PLER-2023,037) and spontaneous operationally tolerant kidney transplanted patients withdrew from immunosuppressive treatments for over one year and with stable creatinine <150 mmol/L and proteinuria 1 g/24h, as previously described [12]. The patients included in the present study differ from those included in the previosuly published study [11]. All donors signed informed consent forms. All procedures were approved by the French “Ministère de L'Enseignement Supérieur et de la Recherche” (MESR) and local ethic committee (“comité de protection des personnes”, CPP n°DC-2011-1399).

Peripheral blood mononuclear cells (PBMC) were isolated from whole blood using Ficoll gradient centrifugation using standard procedures. Cells were stained with CD19 (clone HIB19, BV786, Biolegend) and GZMB (clone QA16A02, PE-Cy7, Biolegend) antibodies and living GZMB^+^ and GZMB^−^ B cells were FACS-sorted on a CYTEK instrument with a 100 μm nozzle. A minimum of 200,000 cells from each subset were centrifuged and genomic DNA was extracted from the cell pellets with a Qiagen kit according to the manufacturer's instructions.

### 2.2 DNA IGH library preparation

IGH FR1-FR4 regions were amplified and sequenced from 500 ng of total genomic DNA. First, genomic DNA was amplified using BIOMED-2 primers [13]. In a second PCR step, Illumina barcodes were incorporated. Primer sequences are listed in Table I. Sequencing was performed on a MiSeq system using the MiSeq Reagent Kit v3 (600 cycles) (Illumina).

**Table 1:** Primers used for IGH sequencing.

PCR1	
VH1-FR1	**5’-**TCGTCGGCAGCGTCAGATGTGTATAAGAGACAGGGCCTCAGTGAAGGTCTCCTGCAAG**-3′**
VH2-FR1	**5′-**TCGTCGGCAGCGTCAGATGTGTATAAGAGACAGGTCTGGTCCTACGCTGGTGAAACCC**-3′**
VH3-FR1	**5′-**TCGTCGGCAGCGTCAGATGTGTATAAGAGACAGCTGGGGGGTCCCTGAGACTCTCCTG**-3′**
VH4-FR1	**5′-**TCGTCGGCAGCGTCAGATGTGTATAAGAGACAGCTTCGGAGACCCTGTCCCTCACCTG**-3′**
VH5-FR1	**5′-**TCGTCGGCAGCGTCAGATGTGTATAAGAGACAGCGGGGAGTCTCTGAAGATCTCCTGT**-3′**
VH6-FR1	**5′-**TCGTCGGCAGCGTCAGATGTGTATAAGAGACAGTCGCAGACCCTCTCACTCACCTGTG**-3′**
JH consensus	**5′-**GTCTCGTGGGCTCGGAGATGTGTATAAGAGACAGCTTACCTGAGGAGACGGTGACC**-3′**
PCR2	
Illumina Forward (P5)	**5′-**AATGATACGGCGACCACCGAGATCTACACXXXXXXXXTCGTCGGCAGCGTC**-3′**
Illumina Reverse (P7)	**5′-**CAAGCAGAAGACGGCATACGAGATXXXXXXXXGTCTCGTGGGCTCGG**-3′**

XXXXXXXX represents the eight nucleotide-long barcode introduced by each PCR2 primer for dual-index sequencing.

Briefly, library preparation began with PCR amplification of 500 ng of extracted DNA using AmpliTaq Gold (Thermo Fisher) and BIOMED-2 primers modified for Illumina sequencing. This first reaction was carried out in a final volume of 50 μL. PCR cycling conditions were: 7 min at 95 °C, followed by 34 cycles of 30 s at 95 °C, 1 min at 60 °C, and 1 min at 72 °C, with a final extension of 10 min at 72 °C.

Then, Illumina adapter and index sequences were added via a second PCR. In this step, 6 μL of the first PCR product was re-amplified using Phusion Taq (New England Biolabs) in a final volume of 25 μL. PCR cycling conditions were: 30 s at 98 °C, followed by 12 cycles of 10 s at 98 °C, 30 s at 62 °C, and 30 s at 72 °C, with a final extension of 5 min at 72 °C.

PCR products were purified using 1.8 × volume of MagPrep purification beads (Merck) and eluted in 30 μL of elution buffer. The final library was prepared by pooling 20 μL from each sample, concentrating the pool using 1.8 × volume of MagPrep beads, and eluting in 40 μL of elution buffer.

The pooled library was size-selected on a PippinHT system (Ozyme) using a 1.5% agarose cassette to recover fragments in the 400–600 bp range. Final quantification was performed on an TapeStation using a D1000 ScreenTape (Agilent). The library was sequenced on a MiSeq instrument using the v3 600-cycle kit, loaded at 20 pM with 30% PhiX control.

#### 2.2.1 Sequence processing and clonotype analysis

Paired-end reads were merged using FLASH software [14], including the concatenation of non-overlapping read pairs. IMGT/HighV-QUEST [15] was used for sequence alignment against the human IG reference database no. 202405-2. Only productive CDR-H3 sequences were retained and grouped into clonotypes, defined at the amino acid level, using the hierarchical Clones function from the SCOPer package [16,17]. Physicochemical CDR-H3 properties were assessed using the Alakazam package [18]. For each repertoire, Hill diversity profiles were generated [19], and the median somatic hypermutation (SHM) frequency was calculated.

#### 2.2.2 Visualization and comparative analyses

Radar plots of Alakazam parameters were generated using the radarchart function implemented in *fmsb* (https://CRAN.R-project.org/package=fmsb). For each variable, normalized values were averaged within the GZMB^–^ and GZMB^+^ groups to allow comparison, and cosine similarity was calculated from these normalized values. Shared clonotypes between repertoires were visualized with Venn diagrams using *ggvenn* (https://CRAN.R-project.org/package=ggvenn) and overlap analyses were performed with functions available in *immunarch* (https://github.com/immunomind/immunarch).

#### 2.2.3 Rarefaction and diversity analyses

Hill diversity profiles and rarefaction analyses were generated using functions implemented in *immunarch*, controlling for the same number of sequences per sample. The number of sequences for subsampling was set according to the smallest sample, and an optimal number of bootstraps was selected for each analysis to ensure robustness.

## 3 Results

### 3.1 Clinical characteristics of the patients

The clinical characteristics of the patients included in this study are summarized in Table II. Spontaneous operationally tolerant kidney transplanted patients withdrew from immunosuppressive treatments for over one year and with stable creatinine <150 mmol/L and proteinuria 1 g/24h, as previously described [12]. Healthy donors were selected and matched for age and sex with the tolerant kidney transplanted patients.

**Table 2:** Clinical data.

	HV1	HV2	TOL1	TOL2
Gender (M/F)	M	M	M	M
Age at the time of sampling (years)	50	60	57	55
Age at the time of transplantation (years)	/	/	33	32
Time post-immunosuppression withdrawal (years)	/	/	2	21
Induction treatment	/	/	NO	NO
Immunosuppression regimen (pre-withdrawal)∗				
Corticosteroids	/	/	YES	YES
Cyclosporine A	/	/	YES	YES
**Mycophenolate mofetil (MMF)**	/	/	YES	YES
Tacrolimus^a^	/	/	YES	YES
Mismatched HLA^b^	/	/	2 (Class 1)	0
**Donor-Specific Antibodies (DSA)**	/	/	-	-
CMV (±)	NA	NA	+	+
EBV (±)	NA	NA	+	+

Abbreviations: CMV Cytomegalovirus; EBV Epstein-Barr Virus; HV Healthy Volunteer; MMF Mycophenolate mofetil; TOL drug-tolerant patient.

a TOL1 was switched from Corticosteroids + Cyclosporine A + MMF to Tacrolimus 3 months post transplantation.

b As the quantity of mismatch for each patient.

### 3.2 GZMB^+^ and GZMB^−^ B cells share the same pattern of V/J gene usages and display comparable CDR3 features

**Fig. 1:**
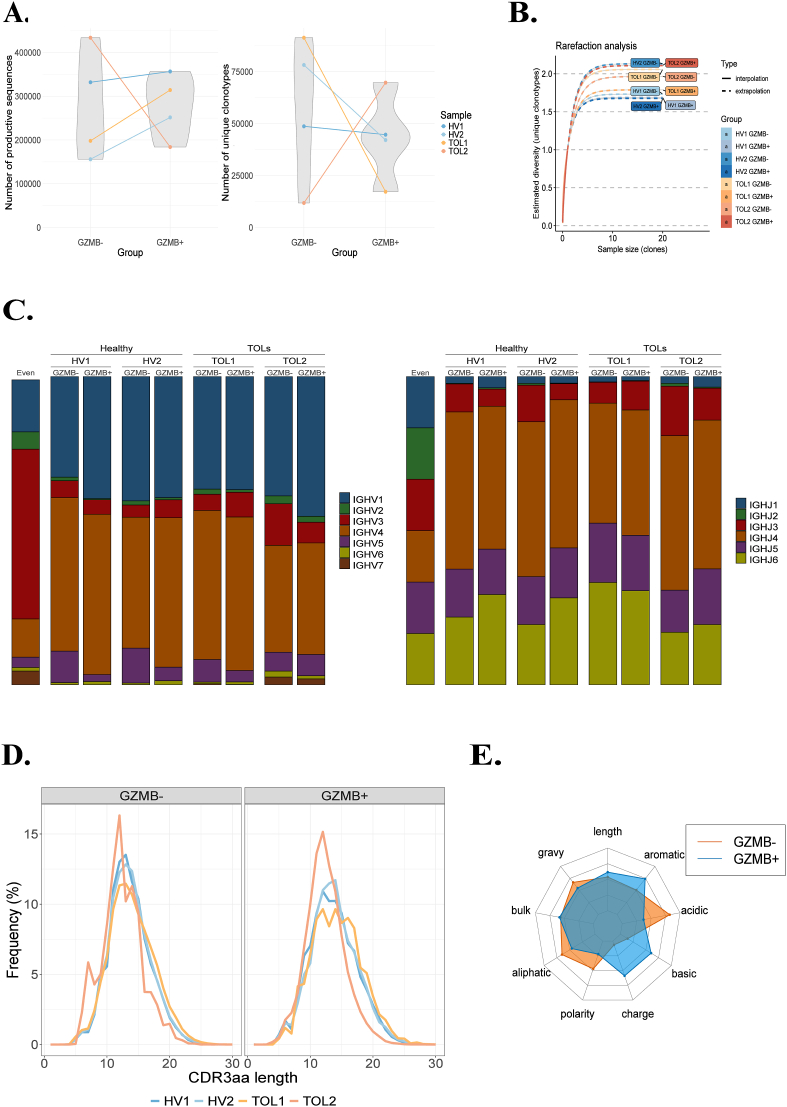
GZMB^+^ and GZMB^−^ B cells display highly similar IGH repertoire features **A.** Unique productive IGH sequences and clonotypes. **B.** Rarefaction curves of clonotype diversity. **C.** IGHV (left) and IGHJ (right) gene usage. **D.** CDR3 length distribution in GZMB^−^ and GZMB ^+^ subsets. **E.** Radar plot of CDR3 physicochemical properties. GZMB: Granzyme B; HV: Healthy Volunteer; TOL: drug-free tolerant patients.

The gating strategy used to sort GZMB^+^ and GZMB^-^ B cell subsets from PBMCs derived from whole blood is shown in Supplementary Fig. 1. IGH sequencing yielded a large number of unique productive sequences, ranging from 155,987 to 433,700 (mean 278,232, standard deviation 96,689) (Fig. 1A). Grouping these productive sequences by hierarchical clustering led to thousands of unique amino acid-defined clonotypes for each sample, ranging from 11,821 to 91,364 (mean 50,489, standard deviation 28,080) (Fig. 1A). Extrapolated rarefaction curves reached a plateau (Fig. 1B), indicating that the total diversity of each sample was approached. In contrast, non-extrapolated curves did not reach a plateau, suggesting that some of the rarest clonotypes were not captured.

Of the parameters available to characterize adaptive immune receptor repertoires, summary statistics about V/J gene usage and CDR3 features are the simplest, yet convey useful information. When paired samples from the same individual were compared, the V/J gene usage of the GZMB^+^ and GZMB^−^ B cell compartments appeared largely superimposable, with a predominant representation of the V1, V4 and J4 gene families (Fig. 1C, Supplemental Fig. 2), as previously reported in the literature for circulating human B cells [20]. The distribution of CDR3 lengths was Gaussian across all samples and did not differ between subgroups (Fig. 1D). In contrast, analysis with the Alakazam package [18] revealed subgroup-specific physicochemical differences in CDR3 sequences, with GZMB^+^ tending to be more basic and charged whereas GZMB^−^ were more acidic. Nevertheless, their overall profiles remained highly comparable (cosine similarity = 0.82), as illustrated by the strong overlap of the radar plot representation (Fig. 1E).

#### 3.2.1 Diversity and mutation analysis suggest shared clonal expansion and evolution of GZMB^+^ and GZMB^−^ B cells

**Fig. 2:**
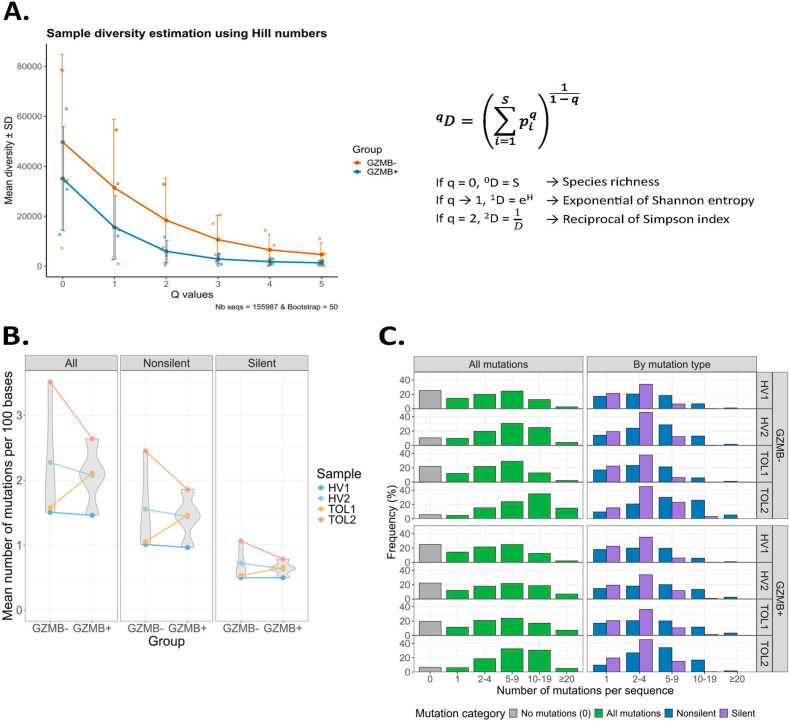
GZMB^+^ and GZMB^−^ B cells exhibit comparable repertoire diversity and somatic hypermutation patterns. **A**. Estimation of diversity through the Hill profiles **B.** Somatic hypermutation (SHM) frequency. Left, all mutations. Middle panel, non-silent mutations. Right panel, silent mutations. **C.** Distribution of mutated and unmutated sequences in paired GZMB^+^ and GZMB^−^ B cell samples. GZMB: Granzyme B; HV: Healthy Volunteer; TOL: drug-free tolerant patients.

To assess the diversity across the whole spectrum of clonotype abundances, we plotted Hill diversity profiles, as previously described by Greiff and al [19]. These profiles, both of which appeared to be polyclonal, followed a similar overall pattern between the GZMB^+^ and GZMB^−^ B cell subsets (Fig. 2A).

Moreover, the SHM was similar for the GZMB^+^ and GZMB^−^ B cells, whether considering the total SHM average frequency or its distribution between non-silent *vs* silent SHM (Fig. 2B). Furthermore, paired samples showed a similar distribution of mutated and unmutated sequences, indicating comparable proportions of naïve and antigen-experienced B cells between the GZMB+ and GZMB− subsets (Fig. 2C).

Together, these results suggest that GZMB^+^ B cells have been selected after experiencing a similar degree of germinal center (GC)-like activation and SHM as GZMB^−^ B cells.

#### 3.2.2 GZMB^+^ B cells do not display any immune convergence between different individuals

**Fig. 3:**
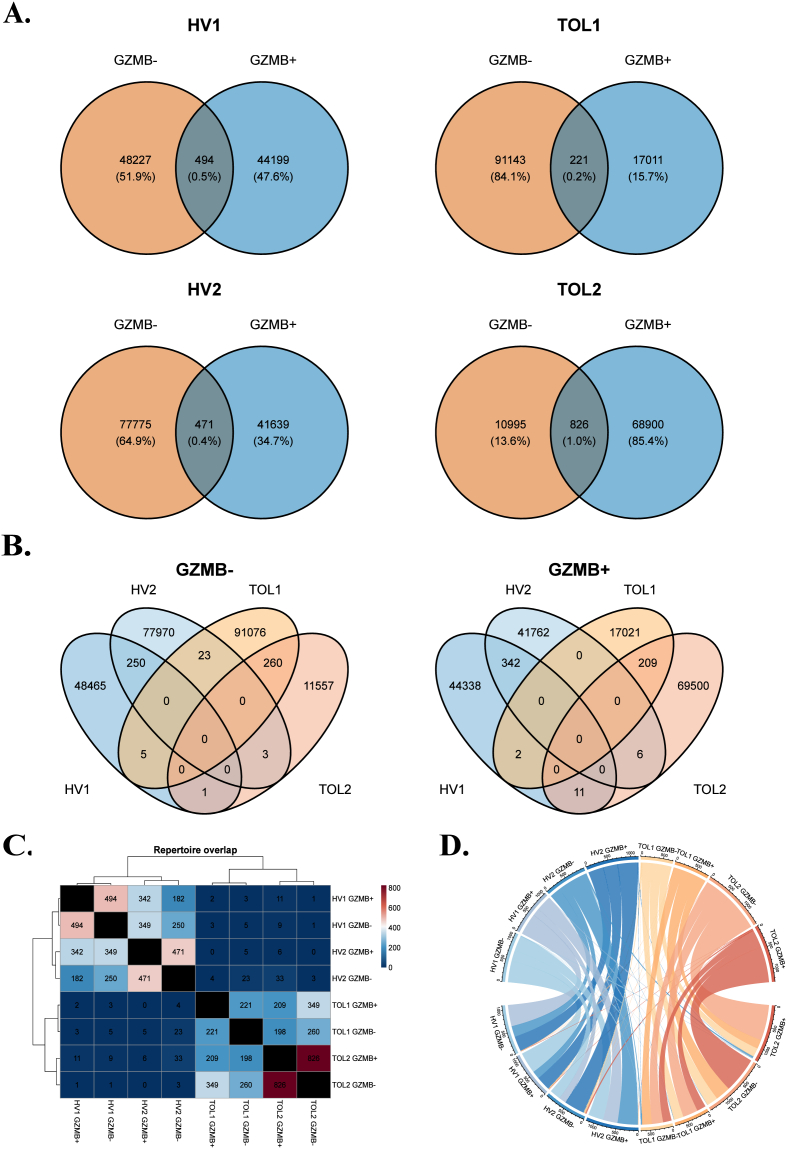
Intra- and interindividual convergence of B cell repertoires in GZMB^+^ and GZMB^−^ subsets A. Intraindividual clonotype overlap. Venn diagrams showing shared and unique clonotypes between GZMB^+^ and GZMB^−^ B cell subsets within each individual (HV1, HV2, TOL1, TOL2). B. Interindividual clonotype overlap. Venn diagrams illustrating shared clonotypes between individuals, stratified by GZMB^−^ and GZMB^+^ subsets. C. Repertoire overlap heatmap. Heatmap representing the magnitude of clonotype sharing across all samples and subsets. D. Circos plot of clonotype sharing. Circos plot visualizing the distribution and overlap of shared clonotypes between individuals and subsets. GZMB: Granzyme B; HV: Healthy Volunteer; TOL: drug-free tolerant patients.

In different immune settings, such as infectious or auto-immune diseases, it has been demonstrated that the immune response may converge and then rely on so-called “public clonotypes”, shared by different, unrelated individuals [21]. To search for such public sequences in our various repertoires, we performed a shared clonotype analysis. As shown in the corresponding Venn diagrams (Fig. 3A), there is a very limited overlap between GZMB^+^ and GZMB^−^ B cells within each sample, and low clonotype sharing between different GZMB^+^ or GZMB^−^ samples from different individuals (Fig. 3B-C). No clonotype was detected in more than two individuals (Fig. 3B). Notably, repertoire overlap appears slightly higher between healthy individuals on one hand and between TOLs on the other, as suggested by the clustering in the heatmap (Fig. 3C) and the connectivity patterns in the Circos plot (Fig. 3D). These results suggest that there is no convergence mechanism shaping the GZMB^+^ B cell repertoire in our patients.

## 4 Discussion

While regulatory T cells are a clearly defined subset of lymphocytes, in particular driven by FOXP3 expression, the definition of Breg cells is fuzzier as these cells are plastic and heterogeneous in nature, with many different subsets described in the literature [1,2].

Among them, we have shown that GZMB^+^ B cells are important players in the immune tolerance of kidney transplants [6].

Mechanistically, the immunoregulatory function of GZMB^+^ B cells is dependent on GZMB itself to at least some extent [7,8] and a contact between the Breg cell and its target. We have previously demonstrated that GZMB expression in *ex-vivo* induced GZMB^+^ B cells from healthy donors is regulated in an autocrine manner by lymphotoxin-α (LTα) [10]. Notably, LTα overexpression was observed only in GZMB^+^ B cells induced from TOLs [11], suggesting that their regulatory and functional properties may be shaped by their microenvironmental context and cellular origin. Furthermore, GZMB^+^ B cells exhibit reduced expression of genes involved in antigen presentation, apoptosis, and inflammatory responses, further supporting their distinct regulatory identity [11].

Although the mechanism of action of GZMB^+^ cells is now better understood, their origin remains elusive. In this study, we used immunoglobulin heavy chain repertoire profiling to investigate their ontogeny. Our results show that GZMB^+^ B cells do not exhibit specific repertoire signatures when compared to their GZMB^−^ counterparts. The V/J gene usage, SHM, CDR3 length distribution and physicochemical characteristics were all highly similar across both populations. Although these observations should be interpreted with caution given the limited cohort size, they suggest that GZMB ^+^ B cells do not develop from B-cell progenitors or immature cells as a distinct sublineage. Instead, they appear to be a functional subset that emerges later from the conventional pool of circulating mature B cells.

Consistently, clonal diversity, as assessed through Hill diversity profiles, indicated that the GZMB^−^ and GZMB ^+^ B cell subsets are similarly polyclonal. SHM analysis revealed comparable hypermutation frequencies, supporting the hypothesis that GZMB^+^ B cells undergo similar GC-like processes to GZMB^−^ cells. Therefore, these data do not support the hypothesis that their acquisition of regulatory function through GZMB expression results from a unique clonal trajectory, but rather are consistent with from contextual cues within their immunological environment.

In addition, the absence of shared GZMB^+^ clonotypes across individuals argues against a common antigen-driven selection. Public clones, which are sometimes observed in response to shared infectious or autoimmune antigens, were virtually absent in the GZMB ^+^ B cell subset. This lack of immune convergence does not support a dominant role for recognition of a common antigen and instead suggests that their emergence may reflect the unique immune histories and microenvironmental signals of each individual. In contrast, other Breg populations have been shown to carry specific repertoires features in mice, such as *Salmonella typhimurium-*induced Lag3^+^ regulatory plasma cells that highly express Vh7, Vh10 and Vh11 [22]. Moreover, graft infiltrating B cells associated with transplant rejection have been shown to be clonally expanded [23].

Such interindividual diversity raises the question of whether GZMB^+^ B cells belong to a specific anatomical or functional compartment. This is consistent with previous studies in humans [24], which identified two primary B cell compartments: a systemic compartment (comprising the blood, bone marrow, spleen and lung tissues) and a mucosal compartment (comprising the gastrointestinal tract). GZMB ^+^ B cells were found in our dataset to resemble conventional circulating B cells. These findings contrast with those for T follicular cell subsets such as T follicular helper (T_FH_) and T follicular regulatory (T_FR_) cells, which carry distinct TCR repertoire characteristics compared to conventional T cells [25].

Treatment-free, transplant-tolerant patients are quite rare. One limitation of the study is that it only included a small number of patients which restricts the generalizibility of the results. It will be important in the future to extend this analysis to more patient cases. However, even with this limited sample size, a clear picture of the IGH repertoire emerges, showing that the diversity of VH usage is quite similar between GZMB^+^ and conventional GZMB^−^ cells.

In conclusion, GZMB ^+^ B cells do not appear to constitute a clonally or antigenically distinct population but rather are consistent with a functional diversification within the peripheral B cell repertoire. Their differentiation is likely influenced by extrinsic factors, possibly including the cytokine milieu, local cell-to-cell interactions, or epigenetic regulation, rather than by antigen-driven clonal selection. These findings emphasize the functional plasticity of human B cells and reinforce the idea that immunoregulatory property can develop within phenotypically and clonotypically diverse B cell pools.

## Authors‘ contributions

SG and NS ran the experiments, analyzed the results and wrote the manuscript; SBST analyzed the results; MCor prepared the libraries; HLM, AD, RD & MG contributed to the experiments, KT provided resources, SB and MCog analyzed the results and contributed to manuscript writing.

## Funding

SG was funded by Fédération Hospitalo-Universitaire CAMIn

## Declaration of competing interest

The authors declare the following financial interests/personal relationships which may be considered as potential competing interests: Cogne, Michel reports financial support was provided by INSERM. If there are other authors, they declare that they have no known competing financial interests or personal relationships that could have appeared to influence the work reported in this paper.

## Data Availability

Repertoire data have been deposited in the European Genome-phenome Archive (EGA) under accession number EGAD50000002452 and in the ZENODO repository under accession number 19333430. Other data supporting the findings of this study are available from the corresponding authors upon reasonable request.
