# Supplementary material for: Granzyme B-expressing regulatory B cells share the same origin as conventional blood B cells
Source: Biochem Biophys Rep. 2026 Apr 17;46:102594. doi: 10.1016/j.bbrep.2026.102594 (PMC13101668; doi:10.1016/j.bbrep.2026.102594)
Supplement: Multimedia component 1 [file mmc1.pdf]

Supplemental figures

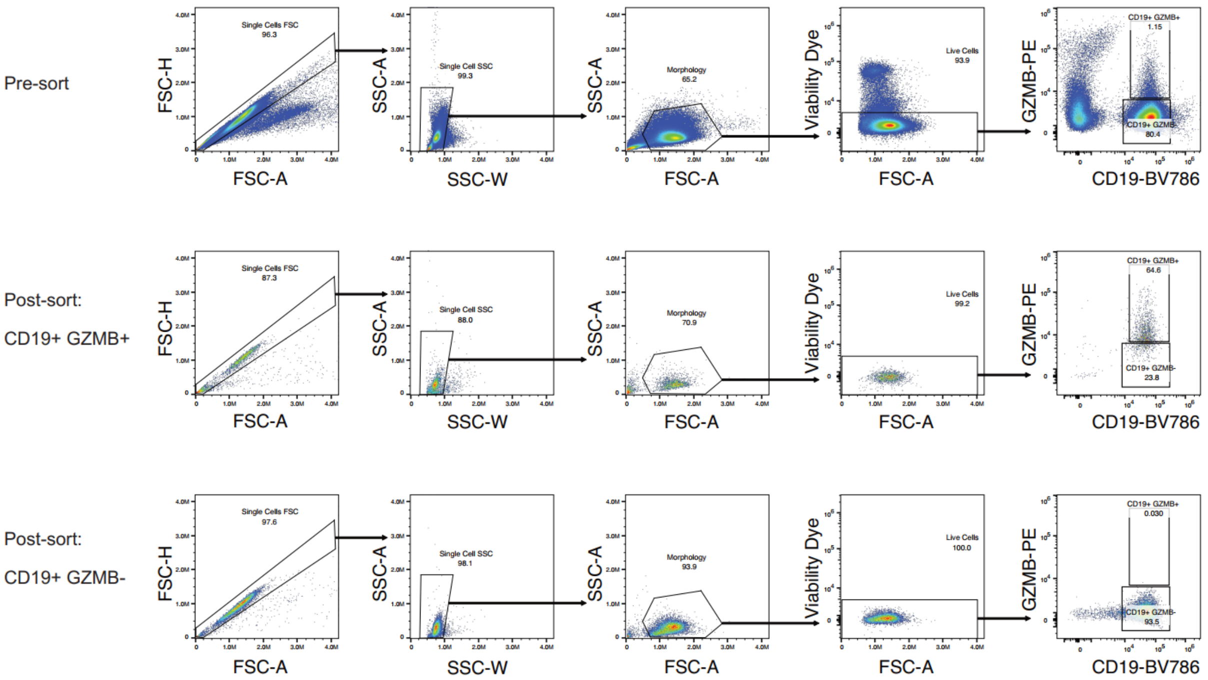

Supplemental Figure 1: Sorting strategy and gating

B cells were pre-enriched from the blood of 4 donors by magnetic sorting and stained for GZMB-PE (Biolegend), CD19-BV786 (Biolegend) and viability Dye (Yellow Live/Dead, Thermo Fisher Scientific). Cells were gated to exclude doublets and cell debris using FSC and SSC parameters, then Viability Dye negative (Live cells), CD19 positive were sorted into two tubes corresponding to GZMB+ and GZMB-. FACS was performed on a Cytek instrument with a 100µm nozzle.

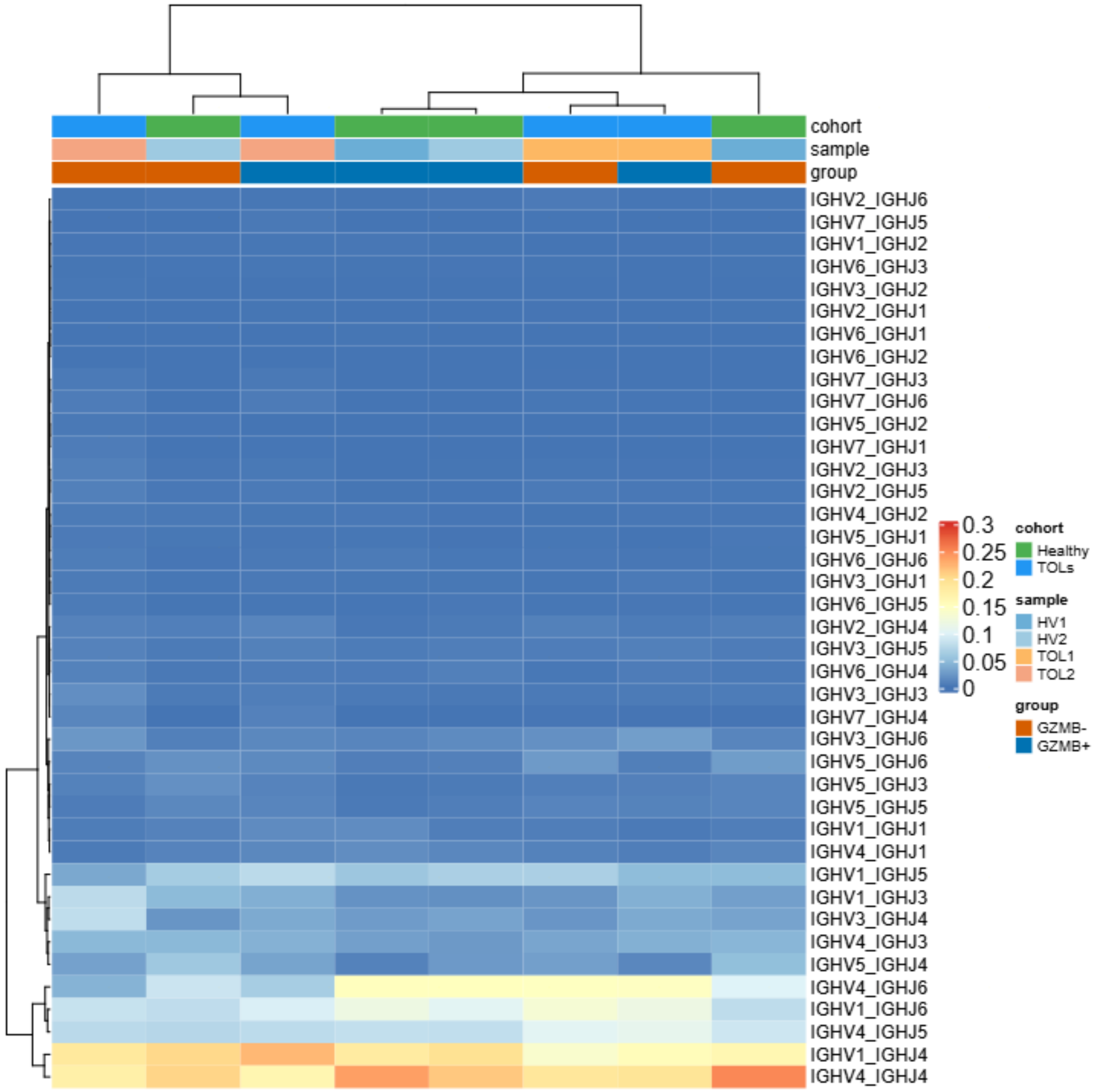

Supplemental figure 2: Heatmap of the V/J gene usage.

Frequencies of V and J gene combinations, at the family level, cannot separate the $GZMB^+$ from the $GZMB^-$ B cells or the healthy donors from the drug-free tolerant patients.
